# Plant Antioxidants in Dry Fermented Meat Products with a Healthier Lipid Profile

**DOI:** 10.3390/foods11223558

**Published:** 2022-11-08

**Authors:** Miriam M. Selani, Ana M. Herrero, Claudia Ruiz-Capillas

**Affiliations:** 1 Institute of Food Science, Technology and Nutrition (CSIC), José Antonio Novais 10, 28040 Madrid, Spain; 2 Center for Nature Sciences, Lagoa do Sino Campus, Federal University of São Carlos (UFSCar), Rod. Lauri Simões de Barros, km 12, SP-189, Buri 18290-000, Brazil

**Keywords:** plant antioxidants, dry fermented meat products, healthier lipid content

## Abstract

Consumers’ perception of meat products has changed in recent years, which has led to an increased interest in healthier meat products. In response to this demand, academia and industry have made efforts to reformulate meat products, especially dry fermented meat products, which are known for their high fat contents, mainly saturated fat. The use of plant or marine oils stabilized in emulsion gels (EGs) or oil-bulking agents (OBAs) as animal fat replacers has been one of the most advantageous strategies to reformulate dry fermented meat products with a healthier lipid content (quality and quantity), but an increase in their polyunsaturated fatty acid content can trigger a significant increase in lipid oxidation, negatively affecting sensory and nutritional quality. The use of antioxidants is the main strategy to delay this deteriorative reaction, but the controversy around the safety and toxicity of synthetic antioxidants has driven consumers and industry toward the use of plant antioxidants, such as phenolic compounds, carotenoids, and some vitamins and minerals. This review provides information about the use of plant antioxidants to control lipid oxidation of dry fermented meat products with healthier lipids.

## 1. Introduction

The development of healthier foods is a current trend in the industry due to growing consumers’ concerns about the relationship between diet and health [1]. Meat products, including dry fermented ones, are among the most studied food categories aiming at a reformulation with a healthier appeal [2,3,4]. Although they provide important elements for human health, such as high biological value proteins, B-complex vitamins (such as B6 and B12), and minerals (iron, etc.) [5], they are also known for their high content of fat, saturated fatty acids, sodium, and some additives whose consumption has been associated with a higher risk of developing some chronic non-communicable diseases [6].

Different strategies for the development of healthier meat products have been studied in order to reduce these last ingredients and enhance the healthy ones [2]. Among the different reformulation strategies, more attention has been paid to optimizing the lipid content (in terms of fat content and lipid profile) of meat products to meet nutritional needs and adhere to health recommendations [4,7,8]. These strategies focus mainly on reducing animal fat and incorporating oils rich in mono-(MUFAs) and polyunsaturated fatty acids (PUFAs), such as some vegetable and marine oils, aiming to increase PUFAs, decrease saturated fatty acids (SFAs), or on the other hand, replace the meat raw material for another with a better lipid profile, and consequently obtain better PUFA/SFA and *n*−6/*n*−3 ratios [9,10,11,12,13,14]. This type of reformulation is especially important in dry fermented meat products that contain high levels of fat (18–65%), mainly saturated fat (12–23.8%) [9,15].

The development of healthier lipid meat products, mainly dry fermented ones, brings benefits from a health point of view; however, these products are more susceptible to lipid oxidation due to their high levels of MUFA and PUFA. The consequences of oxidation are one of the main reasons for consumer rejection of this type of product since it significantly affects their sensory quality and shelf-life, in addition to generating potentially toxic products [5,16,17].

These adverse effects are especially important in dry fermented meat products (such as *sausage chorizo, fuet*, etc.) with a healthier lipid profile, rich in MUFA and PUFA, since the fermentation and drying period during processing and storage before consumption can trigger a significant increase in lipid oxidation, as reported in several studies [18,19,20,21,22].

At an industrial level, the control of this type of oxidation has been carried out in a traditional way with the use of additives, mainly synthetic antioxidants (BHT, BHA, etc.), which allow, in part, to control and retard these processes. Many of these synthetic antioxidants have been critically reviewed regarding their toxicological aspects [23,24,25] and as a result, many countries have banned some antioxidant additives in the manufacture of meat products. In this way, the use of octyl gallate (OG) and dodecyl gallate (DG) as food additives has been banned by the EU [26]. On the other hand, the use of the synthetic antioxidants butylated hydroxyanisole (BHA), butylated hydroxytoluene (BHT), and propyl gallate (PG) in animal-sourced products is limited to 200 mg/kg in lard and other animal fats and dehydrated meats [26], similar to what is allowed in Brazil, where these three antioxidants are limited to fat, at a concentration of 100 or 200 mg/kg, depending on the meat product [27]. In the US, according to the Food and Drug Administration (FDA), BHA and BHT cannot be used as antioxidants in specific meat products [28].

This fact, coupled with the current consumer trend to minimize the additives added to products, has promoted research on the use of vegetables or plant extracts (obtained from fruits, vegetables, spices, herbs, including residues generated during food processing, etc.) rich in bioactive compounds with antioxidant activity that allow them to replace synthetic antioxidants [16,29,30,31].

This is especially important in meat products in which a reformulation is designed with a nutritional and health objective, as is the case of dry fermented meat products with a healthier lipid content (in terms of reducing the fat content and/or improving the fatty acid profile), in which the use of antioxidants is fundamental from the technological and safety point of view. If this antioxidant is also a plant antioxidant, it would even help to further improve the nutritional profile of the final product, controlling not only rancidity, but also acting as an antioxidant for the consumer, making the dry fermented meat product healthier.

Based on this approach, the objective of this review is to focus on the use of plant antioxidants in dry fermented meat products with a healthier lipid content (in quantitative and qualitative terms) due to the importance of these types of products from an innovation point of view.

## 2. Development of Healthy Lipid Content of Dry Fermented Meat Products

There are many strategies for the development of healthier meat products [2], mainly aiming to improve their composition, and among these, the most studied component has been fat and the improvement of the lipid content, together with the reduction in the energy content that this implies [4]. Strategies to address this development are mainly based on the reduction or elimination of unhealthy components (fat, saturated fatty acids, etc.), which may or may not be replaced by a healthy alternative (MUFA, PUFA, etc.) owing to health recommendations [2,8]. All of this is performed while maintaining the same quality criteria demanded for traditional products in terms of their sensory, technological, nutritional, functional, safety, and other characteristics [4,32,33].

These strategies for the development of a healthy lipid content (in quantitative and qualitative terms) can take place at different stages of the production chain, for example, at the level of livestock farms, to obtain the raw material improved in terms of its healthy composition (increase in MUFA or PUFA, antioxidants, etc.) that will later be used in the elaboration of meat products. Another approach can be carried out in the reformulation stage, where components can be incorporated or eliminated. Reformulation strategies are the most common procedures used to develop healthy meat products, and this is the fastest way to modify the composition of the final product [8]. These strategies will depend on different factors, such as the product and the processing applied, the level of substitution, the type of lipid material used, the intended nutritional objective, etc.

In the case of traditional products such as dry fermented meat products with very significant levels of fat (18–65%) [15,32,33], reformulations to partially or totally replace the fat and lipid contents present many difficulties since, in these products, the fat has important technological and sensory functions (appearance, texture, mouthfeel, aroma, etc.) that condition the final acceptability of these meat products [34,35,36]. However, different studies have addressed the possibility of reducing fat in this type of product, using strategies to replace part of the fat with water or a greater amount of lean meat with or without the addition of other ingredients, mainly fibers, oligosaccharides, etc. [37,38,39]. However, this strategy has limitations at the sensory level since it increases hardness due to water loss and does not have a very important impact on the lipid profile of these products. For this reason, different studies have evaluated the use of vegetable or marine oils (olive, soy, fish, etc.) by direct incorporation, encapsulation, pre-emulsion, or structuring [9]. With regard to the direct addition of vegetable oils (olive, soybean) or marine oils (fish oil extracts) to partially replace pork fat in fermented sausages, this procedure allowed lipid optimization at both a quantitative and qualitative level [15,40,41,42]. However, this strategy presented oil retention problems and increased susceptibility to lipid oxidation [43,44,45]. To avoid these inconveniences, other reformulation procedures for fermented products were used, among which those based on lipid structuring processes stand out. Particularly, the strategy based on the replacement of animal fat with structured lipids such as emulsion gels (EGs) or oil-bulking agents (OBAs) offers more attractive applications in the reformulation of health-enhanced dry fermented meat products [18,20,46,47] since they are solid-like plastic materials that have physical and thermal properties similar to those of animal fat but with fewer calories and an improved lipid profile [4,9,18,46]. In this way, a study was carried out using an OBA based on konjac gel with a healthy oil combination as pork backfat replacer in low-fat dry fermented sausages [18,46]. This reformulation strategy improves the PUFA content in this product but decreases the sensory parameters. The combination of oils (vegetables and marines) as animal fat replacers promotes a better approximation to an optimal lipid profile from a health point of view. This strategy has been used by other authors in dry fermented products but in combination with antioxidants extracted from *Melissa officinalis* [48], since the potential problems derived from healthier lipid dry fermented meat products formulations, mainly with higher levels of PUFA and MUFA, is the acceleration of lipid oxidation during storage, which have important implication in the quality and health [18].

## 3. Lipid Oxidation in Dry Fermented Meat Products with Healthier Lipid Profile

Lipid oxidation, together with microbial growth, is one of the main processes that result in the loss of food quality, as the appearance of odors and flavors characteristic of the development of rancidity decreases the acceptability of foods, and the ingestion of non-volatile oxidation compounds can have negative effects on nutritional quality and food safety [29,49]. Meat products are highly susceptible to the occurrence of lipid deteriorative reactions due to their usually high fat content, the presence of pro-oxidants in their composition (heme pigments), and the different processing (grinding, mixing, salting, heating, etc.) used for the preparation of meat products, which favor the contact of lipids with oxygen or the formation of free radicals, increasing the extent of lipid oxidation, also during storage [30].

Lipid oxidation is an autocatalytic process of chain reactions that occurs through the formation of free radicals and consists of three phases: initiation, propagation, and termination. These reactions occur in all saturated and polyunsaturated lipids, but mainly in the latter, in the unsaturated chains of the acyl residues of the triglycerides and can be initiated by different factors (photooxidation, lipoxygenases, metals, etc.). In the autoxidation of lipids, fatty acids react with molecular oxygen in a free radical route, which results in the formation of peroxides and hydroperoxides, the primary oxidation products. These compounds are not considered harmful to the sensory quality of food, as they are odorless. However, they are unstable and can be quickly decomposed into other compounds (secondary oxidation compounds), such as aldehydes, alcohols, hydrocarbons, esters, ketones, and acids, which are responsible for the development of undesirable flavors and odors [16,50]. For the evaluation of the degree of lipid oxidation, chemical methods (thiobarbituric acid reactive substances (TBARS), peroxides, hexanal, etc.) and sensory tests have been used [51,52,53].

The development of lipid oxidation not only affects the sensory quality of meat products through the occurrence of rancidity and off-flavors, but also affects the technological properties of lipids and proteins and reduces their nutritional value due to the loss of essential fatty acids and vitamins. In addition, it affects food nutritional safety through the formation of compounds considered harmful to health, such as some aldehydes, which have been related to cytotoxic, genotoxic, and pro-inflammatory effects [54,55,56,57].

Despite the negative effect of lipid oxidation on sensory quality, in some kinds of products, such as dry fermented meat products, this process leads to the development of their pleasant and typical flavors, which are highly appreciated by consumers [16]. In dry fermented meat products, lipolysis occurs during dry curing. An excess of lipolysis and lipid oxidation could significantly increase the formation of aroma compounds, resulting in rancidity and consumer rejection [58]. Thus, controlling oxidative stability during the processing and storage of reformulated dry fermented meat products enriched with healthier lipids becomes the main challenge due to higher PUFA content, where most studies have reported a significant increase in lipid oxidation of these products.

This increase in lipid oxidation can be explained because the presence of unsaturation in the fatty acid chain reduces the C-H bond strength, making the lipid fraction more susceptible to this process, and causing the oxidation rate to increase very quickly. The dissociation energy of the C-H bond in stearic acid (no double bond) is 99 kcal/mol, while the dissociation energy of this bond adjacent to the double bond in oleic acid and between two double bonds in linoleic acid is 80 and 69 kcal/mol, respectively. As the dissociation energy of the C-H bond decreases, hydrogen is more easily abstracted from the fatty acid, resulting in the generation of a free radical, which can initiate the lipid autoxidation mechanism. For comparison purposes, it is estimated that the susceptibility of linoleic, linolenic, eicosapentaenoic (EPA), and docosahexaenoic acids (DHA) to lipid oxidation is 10, 20, 40, and 50 times higher than that of oleic acid, respectively [59].

These behaviors were observed in the reformulation of dry fermented sausage with the addition of different oils rich in MUFA and PUFA (olive, chia, linseed oil, fish, etc.), where higher susceptibility to lipid oxidation was detected during storage. Increases in thiobarbituric acid reactive substances (TBARS) and hexanal values have been observed in a reformulated dry fermented meat product (“fuet”) with a mixture of olive (high in MUFAs) and chia oils (high in PUFAs) structured in oleogel and emulsion gel [21]. Similarly, a dry fermented sausage with the addition of linseed oil gelled emulsion (16%) rich in *n*−3 fatty acids, as a partial replacer of animal fat, was more susceptible to lipid oxidation during storage [20].

In addition, dry fermented sausages (*chorizo da Pamplona*) reformulated with pre-emulsified fish oil (to increase the PUFAs, EPA, and DHA) as an animal fat replacer in two levels (5.3 g and 10.7 g fish oil/kg), showed that the highest level seemed to accelerate the lipid oxidation process [15]. A konjac matrix containing 10% and 20% of oil, a combination of fish and vegetable oils (flaxseed and olive oils), also showed adverse effects on the oxidative stability of fermented sausage (*chorizo*), which showed TBARS levels four times higher than that of the regular product after 61 days of chilled storage [18].

The detection of rancidity in dry fermented meat products with healthier lipids has also been reported using sensory methods. The reformulation of dry fermented sausage (*chorizo de Pamplona*) enriched with the highest amount of linseed oil gelled emulsion (39.5%) presented changes in odor and taste compared with the control associated with the oxidation process and was evaluated through semi-trained panelists [47]. Similarly, in a sensory analysis with consumers, Solomando et al. [22] evaluated dry-cured sausages enriched with fish oil and reported lower hedonic scores than the control after 4 months of storage, which could be related to changes in the oxidation values.

In order to avoid this oxidation phenomenon in healthier dry fermented meat products, some authors have used microencapsulated oil to prevent the oxidation of omega-3 fatty acids during processing and storage [19,52]. However, even with the use of microencapsulation to incorporate fish oil, the oxidative stability was significantly affected. This unexpected effect has been associated with the fish oil contact with oxygen during the encapsulation process, the temperature used for drying the microcapsules, and their large surface area, allowing greater access of oxygen to *n*−3 fatty acids [60,61,62].

Due to these physicochemical and sensory problems associated with lipid oxidation in dry fermented meat products reformulated to improve the lipid profile, especially those enriched with PUFAs, much work has been performed to control or reduce oxidation, mainly through the use of antioxidants. This is one of the main approaches used by the industry to prolong shelf-life and preserve product quality aiming to avoid consumer rejection of these types of meat products [16].

## 4. Importance of Antioxidants in Meat Products

Antioxidants are mainly added to different meat products for technological and safety reasons in order to prevent lipid oxidation, retard the development of off-flavors, improve color stability and extend the shelf life; however, the use of antioxidants in meat products is also interesting from a nutritional point of view since the meat product could also act as a vehicle for the incorporation of these compounds into metabolism, where it would also exert an antioxidant effect.

An antioxidant can be considered any substance or action procedure that helps to delay or inhibit the oxidation process. Therefore, not only chemical compounds added to the product, but also the use of vacuum or inert gas atmosphere packaging can be considered as such. Regarding chemical compounds, they can be classified as primary and secondary antioxidants, according to their mechanism of action [30].

Primary antioxidants (type I) are those that break the oxidation chain reaction by donating hydrogen to free radicals and generating more stable radicals. In contrast, secondary antioxidants (type II) are those that retard oxidation through other mechanisms, such as metal chelation, regeneration of primary antioxidants, hydroperoxide decomposition, and oxygen scavenging, among others [63]. Moreover, when used in combination with primary antioxidants, they can perform synergistically, i.e., their overall antioxidant activity together is greater than that of the sum of them individually [64].

Both primary and secondary antioxidants are used to control lipid oxidation and extend the shelf life of foods. The most used so far in the industry are the primary antioxidants of synthetic origin, such as butylated hydroxyanisole (BHA, E-320), butylated hydroxytoluene (BHT, E-321), tertiary-butylhydroquinone (TBHQ, E-319), and propyl gallate (PG, E-310) [65]. Among the synthetic secondary antioxidants widely used are ethylenediaminetetraacetic acid (EDTA, E-385), phosphoric acid (E-338), ascorbyl palmitate (E-304(i)), sodium erythorbate (E-316), among others [66]. The use of antioxidants in meat products is regulated by the legislation of each country, which specifies the food in which it can be used, and the amount allowed [28,67]. Other additives used in the meat industry also have an antioxidant function, such as nitrite and nitrate [68].

However, despite the effectiveness of synthetic antioxidants in retard lipid oxidation, the current trend and popularity of clean-label foods [69], which have been linked to products containing natural, harmless, and simple ingredients from the consumer’s point of view [70], have led to an increased interest for natural compounds to replace synthetic additives. In addition, consumers are willing to pay more for clean-label products and foods with natural additives [71,72].

## 5. Compounds/Ingredients from Plants with Antioxidant Action

**Figure 1:**
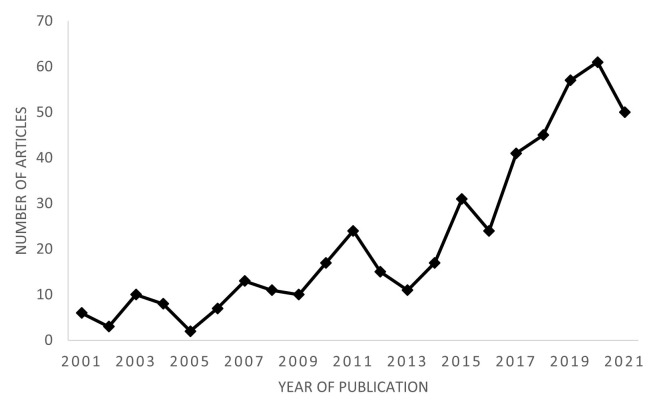
Number of articles published on natural or plant antioxidants in meat and meat products in the last 20 years (extracted from Web of Science).

Given this current trend of clean-label foods, extensive research has been conducted on the identification and characterization of plant-based antioxidant substances [73,74,75,76,77]. As a consequence, studies on the application of these substances as replacers of conventional synthetic antioxidants in meat and meat products have increased in recent years, as shown in Figure 1.

Due to the high content of bioactive compounds with antioxidant activity in plants, the most studied plant antioxidant sources in meat products are fruits, vegetables, spices, and herbs [78,79,80,81]. Seeking the rational use of plant materials, strategies for reusing agro-industrial residues as sources of bioactive compounds with antioxidant activity, following the concept of circular economy, have also been extensively studied [82]. The transformation of agro-industrial residues into high-value-added products/ingredients to be used in animal feed or directly in food products is an interesting option to be exploited, as it reduces waste disposal in the environment, promotes sustainability, and represents a new business opportunity for the industry [74,83,84].

It is important to emphasize that although various plant ingredients/compounds have been reported to present antioxidant activity, their use in food should consider some important factors, such as safety for consumption, stability to processing and storage conditions, solubility, efficacy at low concentration, availability, compatibility with the food matrix (mainly regarding sensory properties) and regulatory guidelines [85,86]. Regarding the latter, only a few compounds obtained from plants are currently allowed for use as food additives in meat products by the European Regulation 1333/2008 [67] (Table 1), which could confer antioxidant action.

The antioxidant activity of plant materials is mainly due to the presence of phenolic compounds, but it may also be linked to other substances, such as carotenoids, some vitamins (C and E), and minerals (selenium and zinc), as described below.

### 5.1. Phenolic Compounds

Phenolic compounds comprise a large class of natural substances produced as plant secondary metabolites with different chemical structures and activities. They have an aromatic ring and a benzene ring with one or more hydroxyls as functional groups, including functional derivatives (glycosides, esters, methyl esters, etc.), which are responsible for their antioxidant properties [87]. They are present in the vast majority of fruits and vegetables, as well as in cereals, roots, and leaves, usually produced as a defense mechanism of plants, which are among the substances with the most studied antioxidant activity in foods [88,89]. Considering the structural variations between phenolic compounds, they are divided into different classes, such as flavonoids, phenolic acids, tannins, lignans, and coumarins [85,89], in which flavonoids and phenolic acids stand out. In this sense, the health benefits resulting from the antioxidant action of phenolic compounds against oxidative stress diseases have been highlighted by several studies [89].

Flavonoids are a class of phenolic compounds with different structures, found in a wide range of fruits, mainly berries, in which anthocyanins are predominant, and vegetables, such as broad bean pod, black olive, red onion, spinach, and shallot [90]. These compounds have shown important functions, such as anti-oxidative, anti-inflammatory, anti-mutagenic, and anti-carcinogenic activities, with potential health benefits [91]. Flavonoids are divided into different subclasses based on their structure, such as anthocyanins, flavones, flavanones, and isoflavonoids, chalcones, neoflavonoids, and flavanonols. Anthocyanins are natural pigments that impart color to fruits, plants, and flowers and include the subclasses cyanidin, delphinidin, malvidin, pelargonidin, and peonidin. Flavones are important bioactive flavonoids found in fruits, leaves, and flowers as glucosides. They have a double bond between positions C2 and C3 and a ketone at position C4 and include the subclasses apigenin, tangeretin, baicalein, and rpoifolin. Flavanones or dihydroflavones have the C-ring saturated between positions 2 and 3. These flavonoids are subdivided into hesperitin, naringin, naringenin, hesperidin, and eriodictyol. Isoflavonoids are a diverse subgroup of flavonoids that include genistin, genistein, daidzein, glycitein, and daidzin. Chalcones are a class of flavonoids that lack the C-ring in their structure. This class includes the subclass philoridzin, arbutin, phloretin, and chalconaringenin. Neoflavonoids are a class of polyphenolic compounds that have a 4-phenylchromen backbone with no hydroxyl group substitution at position 2. Flavanonols, also referred to as dihydroflavonols or catechins, are the 3-hydroxy derivatives of flavanones [91]. Due to the hydroxyl groups attached to ring structures, flavonoids have the ability to act as reducing agents, superoxide radical scavengers, hydrogen donators, singlet oxygen quenchers, and metal chelators [92]. Catechin, catechin-gallate, quercetin, and kaempferol represent some of the most important compounds of this class [92].

Phenolic acids are hydroxy derivatives of cinnamic acid (e.g., p-coumaric, caffeic, ferulic, and sinapic acids) and benzoic acid (e.g., gallic acid, p-hydroxybenzoic acid, protocatechuic acids, vanillic acid, and syringic acid) [93]. The antioxidant activity of these compounds is related to their ability to chelate pro-oxidant metal ions and scavenge free radicals [92], which is greater in hydroxycinnamic acids than in hydroxybenzoic acids [94]. Good sources of phenolic acids are blueberry, cherry, apple, pear, grapefruit, orange, peach, lemon, potato, lettuce, spinach, coffee beans, tea, and coffee [95].

Polyphenols from different plant origins, including agro-industrial residues, have been extensively studied as antioxidants and antimicrobials in meat products [81,96,97] and also as a substitute for vitamin E or supplement in animal feed [29,98,99].

In addition to their antioxidant properties, these compounds have anti-inflammatory, anti-aggregating, antimicrobial properties, among others. They have also been shown to play an important role in preventing numerous cardiovascular diseases, strokes, high blood pressure, different types of cancer, neurodegenerative, inflammatory, eye diseases, obesity, diabetes, osteoporosis, etc. [100,101].

### 5.2. Carotenoids

Carotenoids are fat-soluble natural pigments synthesized by plants, algae, as well as some bacteria and fungi [102], but found predominantly in fruits and vegetables. They are classified into two groups, according to their chemical composition: carotenes (hydrocarbon carotenoids), such as β-carotene and lycopene, and xanthophylls (oxygenated carotenoids), such as lutein, capsanthin, zeaxanthin, canthaxanthin, and astaxanthin [103]. They are considered effective antioxidants mainly due to their action as singlet oxygen physical quenchers [104]. In this mechanism, carotenoids deactivate singlet oxygen to the ground state (triplet oxygen) by energy transfer. This action produces excited carotenoids that dissipate the acquired energy to the environment and return to their original state, allowing them to quench more radical species [92,105]. This singlet oxygen quenching ability increases with the increasing number of conjugated double bonds in the carotenoid chain [96], and for this reason, β-carotene and lycopene, which have 11 conjugated double bonds, are known to be more efficient singlet oxygen quenchers than lutein (10 conjugated double bonds) [106]. Carotenoids are also potential scavengers of peroxyl radicals, resulting in the formation of resonance-stabilized radical adducts and, consequently, leading to a disruption in the propagation of lipid oxidation [107]. They can be found in colorful edible plants. Yellow-orange vegetables and fruits, such as carrots and apricots, are generally rich in carotenes; orange fruits, such as mandarins and papaya, are good sources of β-cryptoxanthin. Lutein and β-carotene are commonly found in dark green vegetables, such as spinach and kale. Tomatoes and their products are sources of lycopene (red color) [108,109].

In addition, some carotenoids have provitamin A activity, so they can be converted to vitamin A in our body [94], which is an essential micronutrient for maintaining vision, promoting cell growth, and enhancing the immune system [110]. Moreover, they act as antioxidants in the cell by participating in the neutralization of reactive oxygen and nitrogen species produced as part of the cellular metabolism, acting as protective agents against various diseases, such as cancer and cardiovascular diseases [111].

### 5.3. Vitamin E from Plants

Plants are sources of vitamin E, found mainly in vegetable oils (soybean, sunflower, corn, walnut, cottonseed, palm, and wheat germ oils) and nuts (hazelnut and almonds) [112,113], which may have their extracts used as an interesting antioxidant. Vitamin E is a group of eight lipid-soluble isoforms divided into two classes: tocopherols (α-, β-, γ-, δ-tocopherols), which have a saturated side chain, and tocotrienols (α-, β-, γ-, δ-tocotrienols), which have an unsaturated side chain. The four isoforms of each class (α-, β-, γ-, δ) vary in the number of methyl groups on the chroman ring [114]. Vitamin E is an antioxidant extracted from nature common in the food industry and animal feed [115,116]. Tocopherols and tocotrienols are known as natural antioxidants for lipids, with α-tocopherol being the only one that meets human vitamin E requirements [117]. These compounds act as efficient free radical scavengers through the donation of a hydrogen atom to peroxyl radicals [92]. Thus, vitamin E prevents the propagation of the autocatalytic reaction since peroxyl radicals react 1000 times faster with vitamin E than with PUFA. The resulting tocopheroxyl or tocotrienoxyl radical can be reduced by vitamin C and regenerated to the corresponding vitamin E form, reinstating its antioxidant action [103,117,118]. The highest antioxidant efficacy among the α, β, γ, and δ isoforms, as well as among tocotrienols and tocopherols, is controversial in the literature [119], since the nature of the substrate modifies the antioxidant activity [120].

At the metabolic level, vitamin E has antioxidant, anti-inflammatory, immunoregulatory and neuroprotector actions, helping to prevent neurological and chronic diseases, especially those related to oxidative stress, such as atherosclerosis and cancer [121,122].

### 5.4. Vitamin C from Plants

Vitamin C is a water-soluble antioxidant constituted by L-ascorbic acid and its reduction product, L-dehydroascorbic acid [123]. The main food sources of vitamin C are fruits, mainly citrus fruits, and vegetables, such as green leafy vegetables, potatoes, broccoli, Brussels sprouts, tomatoes, and peppers [124]. It acts as an electron donor, scavenging free radicals in aqueous media, such as superoxide radical anion, hydroxyl radical, hydrogen peroxide, and singlet oxygen, thus stabilizing the reactive species. It can potentially prevent peroxidation initiation by eliminating peroxyl radicals [64]. Despite not being a direct radical scavenger in lipid media, vitamin C can indirectly act in the protection of lipids due to its synergistic action with vitamin E [103]. As previously mentioned, at the lipid-aqueous interphase, ascorbic acid donates a hydrogen atom to tocopheroxyl or tocotrienoxyl radicals, regenerating the active antioxidant form of vitamin E [118].

Besides its antioxidant function, vitamin C acts on collagen metabolism, which is important for tissue growth and regeneration, in addition to enhancing iron absorption and availability [125]. Due to this last function, its use in meat products, which are rich in highly available iron, is very important.

This vitamin, in addition to being an antioxidant, has also been used as an antimicrobial together with vitamin E in hot dogs [126].

### 5.5. Minerals from Plants

Selenium and zinc are the most important minerals having an antioxidant role. They do not directly act on free radicals but act as a cofactor of antioxidant enzymes [92,127].

Selenium is an essential trace mineral found in food of animal origin (meat and meat products, offal, fish, dairy products), in vegetables, such as cereals (rice and wheat) and nuts (mainly Brazil nuts), as well as in mushrooms [128]. This mineral is an indispensable component of glutathione peroxidase (GPx), an enzyme involved in the decomposition of both hydrogen peroxides and hydroperoxides produced during lipid oxidation [64,129]. It is also an essential part of thioredoxin reductase, which reduces lipid peroxides [130] and is involved in recycling vitamin C [131]. For these functions, selenium is one of the main antioxidants studied in dietary supplementation of animals to control lipid oxidation in meats. For human health, selenium is an essential element for the antioxidant organism that appears to inhibit cell proliferation and prevent cell damage, having a protective effect against cancer [132]. This effect is based on the relationship between antioxidant enzymes in the human body and Se. Low levels of trace elements, such as selenium, along with an insufficient concentration of antioxidant enzymes, may be an important contributing factor to oxidative stress, which can induce biological damage. Protein oxidation is associated with a number of pathologies and aging [133], such as endothelial dysfunction in pre-eclamptic/eclamptic mothers [134], epithelial inflammation and epithelial ovarian cancer [135], and Alzheimer’s disease [136]. Selenoproteins, as an antioxidant, can also prevent cell damage caused by cellular protein oxidation. Se acts together with vitamin E and is a component of selenoproteins, thus playing an important role in both the first and second lines of antioxidant defense [133]. This mineral, in addition to having antioxidant and antimicrobial activity in food [137], has also been used to enrich meat products [2].

Zinc is also an essential element of the antioxidant system in animals, whose dietary supplementation may increase the endogenous antioxidant and antimicrobial capacity [138,139]. Whole grains, nuts, and beans are plant sources of this mineral [140]. It acts as a cofactor of the antioxidant enzyme superoxide dismutase that converts superoxide radical anion into hydrogen peroxide [103]. This mineral induces the production of metallothioneins (intracellular metal-binding proteins) that can scavenge hydroxyl radicals [141]. It also competes with iron and copper for specific binding sites [142], thus decreasing the production of free radicals. Regarding its metabolic functions, in addition to its antioxidant properties, zinc has anti-inflammatory action, participates in the maintenance of the skin and membranes, and plays an important role in the immune system [143].

## 6. Application of Plant Antioxidants in Dry Fermented Meat Products with Healthier Lipid Profile

There are many studies on the application of plant antioxidants in meat products. In addition, plant antioxidants have also been used in animal feed, with the aim of finding healthier raw meats for the production of healthier meat products; however, few studies have focused on the use of plant antioxidants (in animal feed or directly in the product) to control the lipid oxidation of dry fermented meat products with a healthier lipid profile, despite the adverse effects of PUFAs on the oxidative stability of these meat products and the current trend of using more natural ingredients to replace traditional additives.

Plant antioxidants were used in animal feed to obtain healthier meat as raw material for the production of healthier lipid meat products with improved oxidative stability. Mairesse et al. [144,145] evaluated dry-cured hams manufactured with meat from pigs fed diets with linseed and two different natural antioxidants: (1) hydrophilic polyphenols, mainly resveratrol (3 kg/t) and (2) extracts from rosemary, citrus fruits, grapes, and *Tagetes* sp. (2 kg/t). The diet supplementation with linseed resulted in dry-cured hams with *n*−6/*n*−3 ratios lower than four and high contents of α-linolenic acid. The addition of plant antioxidants to the animals’ diet reduced the lipid oxidation of dry-cured hams rich in *n*−3 by approximately half of the levels found in the control (diet without antioxidants). Despite this fact, the sensory characteristics of the dry-cured hams were not affected.

Three agro-industrial residues source of phenolic compounds (lyophilized beer residue extract, aqueous extract of chestnut leaves, and ethanolic extract of peanut skin, all at a concentration of 2000 mg/kg product) were evaluated as antioxidants in a Spanish dry fermented sausage with partial substitution of pork backfat by microencapsulated fish oil (rich in EPA and DHA) stabilized in a konjac matrix [145]. These authors reported that there was no significant difference in TBARS levels between the control sausage and those with antioxidants, but the hexanal and the total aldehyde content were reduced in samples with the addition of the three residue extracts, suggesting their protective effect against lipid oxidation.

García-Íñiguez de Ciriano et al. [48] evaluated the partial replacement of pork back fat by an emulsion containing a mixture of linseed/algae oil in dry fermented sausages (*chorizo de Pamplona*) and used a lyophilized aqueous extract of *Melissa officinalis* (686 ppm) as an antioxidant to control lipid oxidation. The incorporation of linseed–algae oil in dry fermented sausage increased the linolenic acid, EPA, and DHA contents and decreased the *n*−6/*n*−3 ratio, favoring the oxidation of these meat products. The lipid oxidation after the ripening process did not show significant differences between products, and the incorporation of linseed–algae oil did not affect the sensory quality of the sausages. However, when this product was evaluated during 90 days of refrigerated storage, the TBARS value of the control (without plant antioxidant) was three times higher than that of the *chorizo de Pamplona* with improved lipid profile and addition of plant extract, showing the effectiveness of *Melissa officinalis* as an antioxidant [146]. This behavior could be attributed to the phenolic components present in these plant extracts [147,148].

In a similar study, García-Íñiguez de Ciriano et al. [149] investigated the capacity of another plant extract (340 ppm of lyophilized aqueous extract of *Borago officinalis* leaves) as an antioxidant in *chorizo de Pamplona* enriched with linseed oil. The lipid reformulation increased the α-linolenic acid content (9-fold the values found for the control), and the addition of *Borago officinalis* extract decreased the peroxide, TBARS, and hexanal values, reflecting an improvement in lipid profile and oxidative stability of the sausages. This delay in lipid oxidation may be due to the phenolic content and antioxidant activity found in the *Borago officinalis* extract. Although the lipid oxidation indices were higher in the control sausages, no significant difference was found between the samples in the quantitative descriptive analysis, indicating that in the sensory evaluation, the panelists did not detect differences in relation to the intensity of lipid oxidation.

Pavlík et al. [150] also studied the incorporation of microencapsulated linseed oil in a dry fermented sausage (Poličan) and a hot smoked dry sausage (Vysočina), with and without rosemary extract. These authors reported an increase in PUFA content and a decrease in the *n*−6/*n*−3 ratio in both sausages with microencapsulated linseed oil, which also showed higher TBARS levels compared to the control. However, when rosemary extract (0.3 g/kg product) was used in combination with microencapsulated linseed oil, a significant decline in lipid oxidation levels was observed in the meat products. This effect is certainly related to the presence of several antioxidant components in rosemary extracts, which belong to the class of phenolic compounds [151].

## 7. Conclusions

The use of structured lipids such as emulsion gels or oil-bulking agents containing certain vegetable or marine oils as animal fat replacers is a promising strategy to develop healthier lipid meat products in line with health recommendations (higher MUFAs and PUFAs, among others) and may help to overcome the negative effects of animal fat replacement on sensory and technological properties, especially in dry fermented meat products, which are high in fat. However, although the lipid profile of these meat products is nutritionally improved due to the higher PUFA content, studies have reported a significant increase in lipid oxidation during storage, which has negative implications for sensory quality and food safety.

The main approach to control lipid oxidation in meat products, especially those reformulated to obtain a healthier lipid profile, is the use of antioxidants. Due to the possible toxicity of synthetic antioxidants, the current trend is to minimize the use of this food additive by replacing it with plant compounds that have antioxidant activity, such as phenolic compounds, carotenoids, vitamins C and E, and minerals (mainly selenium and zinc). Consequently, studies on the application of these substances as substitutes for synthetic antioxidants in animal feed or directly in meat products have increased in the last 20 years.

Studies have confirmed that the use of plant antioxidants can extend the shelf life of dry fermented meat products with healthier lipid content (in terms of reducing the fat content and/or improving the fatty acid profile) by delaying lipid oxidation, in addition to maintaining their sensory characteristics. However, it is important to emphasize that there are limitations to their industrial application, as EU regulations allow the use of only a few compounds obtained from plants as food additives in meat products.

## Figures and Tables

**Table 1:** Compounds/ingredients obtained from plants allowed for use as food additives in meat products by the EU that could confer antioxidant action.

Additive	Maximum Level (mg/L or mg/kg)	Product
Meat preparations		
Curcumin	20	Only merguez type products, *salsicha fresca*, *butifarra fresca*, *longaniza fresca* and *chorizo fresco*
Annatto bixin	20	Only *breakfast sausages* with a minimum cereal content of 6% and *burger meat* with a minimum vegetable and/or cereal content of 4% mixed within the meat
Annatto norbixin	20	Only *breakfast sausages* with a minimum cereal content of 6% and *burger meat* with a minimum vegetable and/or cereal content of 4% mixed within the meat
Paprika extract	10	Only merguez type products, *salsicha fresca*, *butifarra fresca*, *longaniza fresca*, *chorizo fresco*, *bifteki*, *soutzoukaki* and *kebap*
Beetroot red	*quantum satis*	Only merguez type products, *salsicha fresca*, *butifarra fresca*, *longaniza fresca* and *chorizo fresco*
Ascorbic acid	*quantum satis*	Only *gehakt,* prepacked preparations of fresh minced meat and meat preparations to which other ingredients than additives or salt have been added
Non-heat-treated meat products *		
Curcumin	20	Only sausages
	*quantum satis*	Only *pasturmas*
Carotenes	20	Only sausages
Annatto bixin	20	Only *chorizo sausage*, *salchichon*, *pasturmas* and *sobrasada*
Annatto norbixin	20	Only *chorizo sausage*, *salchichon*, *pasturmas* and *sobrasada*
Paprika extract, capsanthin, capsorubin	10	Only sausages
Beetroot Red, betanin	*quantum satis*	Only sausages
Extracts of rosemary	100	Only dried sausages
	15	Only meat with a fat content not higher than 10%, excluding dried sausages
	150	Only meat with a fat content higher than 10%, excluding dried sausages
	150	Only dehydrated meat
Heat-treated meat products		
Curcumin	20	Only sausages, pâtés and terrines
Carotenes	20	Only sausages, pâtés and terrines
Annatto bixin	20	Only sausages, pâtés, terrines and *luncheon meat*
Annatto norbixin	20	Only sausages, pâtés, terrines and *luncheon meat*
Paprika extract, capsanthin, capsorubin	10	Only sausages, pâtés and terrines
Beetroot Red, betanin	*quantum satis*	Only sausages, pâtés and terrines
Ascorbic acid	*quantum satis*	Only *foie gras*, *foie gras entier*, *blocs de foie gras/Libamáj*, *libamáj egészben*, *libamáj tömbben*
Extracts of rosemary	15	Only meat with a fat content not higher than 10%, excluding dried sausages
	150	Only meat with a fat content higher than 10%, excluding dried sausages
	100	Only dried sausages
	150	Only dehydrated meat

*Merguez type products:* traditional French preparations obtained by grinding and mixture from meat and fat of animals, ox(beef) and /or sheep for the most typical. The red color and the spicy taste are characteristic”. *Salsicha fresca*: fresh sausage meat products, prepared with minced meat, seasoned with salt, pepper, and other spices and stuffed in natural or artificial casings. Traditional in Portugal. *Longaniza fresca and butifarra fresca*: Meat preparations made from fresh comminuted meat, fat and/or edible meat offal of farmed animal, seasoned with salt, pepper, and other spices and additives, mixed and stuffed in natural or artificial casings. Traditional from Spain. *Breakfast sausage:* meat preparation from Ireland and the United Kingdom. In this product, the meat is minced in such a way that the muscle and fat tissues are completely dispersed, so the fibers make an emulsion with the fat, giving the product its typical appearance. Examples of other ingredients include cereals, spices, and herbs. *Burger meat*: with a minimum vegetable and/or cereal content of 4% mixed within the meat. *Bifteki:* product from Greece and Cyprus, produced from minced meat >50%, with the addition of bread products and various vegetables of at least 8%, seasonings, other foodstuffs, and permitted food additives. *Soutzoukaki:* product from Greece and Cyprus, produced from minced meat >50%, with the addition of bread products and various vegetables of at least 8%, seasonings, other foodstuffs, and permitted food additives. *Kebap:* product from Greece and Cyprus, prepared from beef and/or lamb minced meat >75%, with the addition of bread products and various vegetables and other foodstuffs. *Gehakt:* minced meat, not being a separator meat, with a fat content of no more than 25%, originating from one or more animals for slaughter, which, by means of chopping, grinding, or other means, is more or less reduced in size; kneadable in such a way that it can be reshaped into different forms. Traditional from the Netherlands. *Pasturmas*: a strongly dehydrated fermented-cured chopped meat product. Produced mainly from beef meat (as well as from sheep and goat meat), cut into strips οf 12–20 cm width, thickness of about 5–8 cm and of 40–50 cm long. Traditional from Turkey and Armenia. *Salchichón y chorizo:* sausage meat products made of pig minced meat and fat (or other animals), cured, seasoned with pepper and paprika, respectively, and other spices, stuffed in natural or reconstituted artificial casings, fermented and smoked, subjected to maturing and drying for at least 30 days at controlled temperature. Traditional from Spain. *Sobrasada*: sausage meat products made of minced pig meat and fat (or other animals), seasoned with paprika, salt and spices, mixed to obtain a spreadable paste, encased in natural or reconstituted artificial casings, followed by maturing and drying. Traditional from Spain. *Luncheon meat:* emulsion-type cured meat product made of chopped or comminuted beef or poultry that is sterilized by heat. *Foie gras*: liver product that consists of pieces of lobes of fattened goose or duck liver and a seasoning. Traditional in France. *Foie gras entire*: liver product that consists of a whole fattened liver or one or more lobes of fattened goose or duck liver and a seasoning. Traditional in France. *Le bloc de foie gras*: liver product that consists of reconstituted foie gras of goose or duck and a seasoning. Traditional in France. *Libamáj egeszben* (natural goose liver): liver product of which at least 90% of it consists of one or more lobes of fattened goose liver and is only salted and preserved by heat treatment. Traditional from Hungary. *Libamáj tömbben* (block of goose liver): liver product placed into a mold that may be lined with a slice of lard; it contains a minimum of 85% goose liver in the liver paste section without the fat and together with the visible liver pieces; it is preserved by heat treatment. Traditional from Hungary. * Dry fermented meat products are in the food category “non-heat-treated meat products”.

## Data Availability

Not applicable.
